# Supplementary material for: Oscillatory surface rheotaxis of swimming E. coli bacteria
Source: Nat Commun. 2019 Jul 31;10:3434. doi: 10.1038/s41467-019-11360-0 (PMC6668461; doi:10.1038/s41467-019-11360-0)
Supplement: Supplementary file 2 — Supplementary Information [file 41467_2019_11360_MOESM2_ESM.pdf]

Oscillatory surface rheotaxis of swimming *E. coli* bacteria – Supplemental Information

Arnold J. T. M. Mathijssen,[1,2] Nuris Figueroa-Morales,[3,*] Gaspard Junot,[3] Éric Clément,[3] Anke Lindner,[3,†] and Andreas Zöttl[2,3,4,‡]

[1] *Department of Bioengineering, Stanford University, 443 Via Ortega, Stanford, CA 94305, USA*
[2] *Rudolf Peierls Centre for Theoretical Physics, University of Oxford, 1 Keble Road, OX1 3NP, UK*
[3] *PMMH, UMR 7636 CNRS-ESPCI-PSL Research University, Sorbonne University, University Paris Diderot, 7-9 quai Saint-Bernard, 75005 Paris, France*
[4] *Institute for Theoretical Physics, TU Wien, Wiedner Hauptstraße 8-10, Wien, Austria*

* Present address: Department of Biomedical Engineering, The Pennsylvania State University, University Park, PA 16802, USA
† anke.lindner@espci.fr; joint corresponding authors
‡ andreas.zoettl@tuwien.ac.at; joint corresponding authors

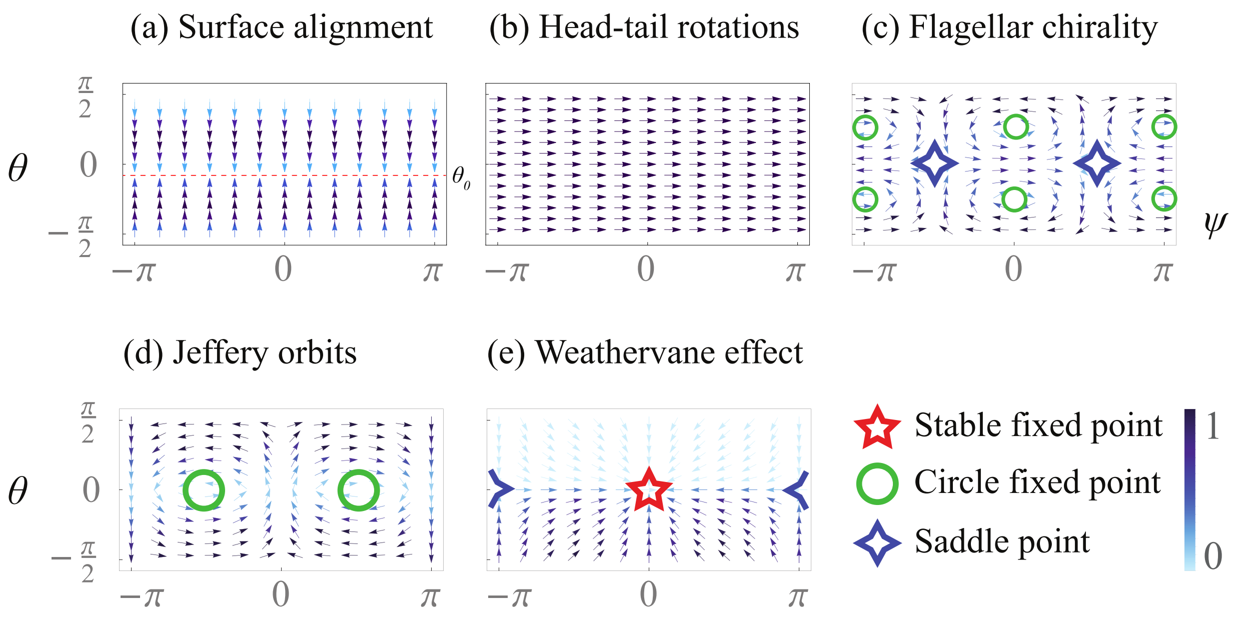

Supplementary Figure 1. Individual reorientation dynamics for bacteria undergoing surface rheotaxis. (a) Wall-induced alignment due to hydrodynamic and steric interactions enable bacteria to swim parallel to the wall in the absence of flow. (b) Counter-rotation of head and flagellar bundle leads to swimming in circles. (c) Reorientation in flow due to chirality of flagella. (d) Jeffery reorientation of an elongated bacterium in shear flow. (e) Anchoring of cell body close to the wall orients the bacterium upstream. The color code indicates the angle-dependent strength of the individual reorientation contributions, and stable fixed points, circle fixed points and saddle fixed points are marked.

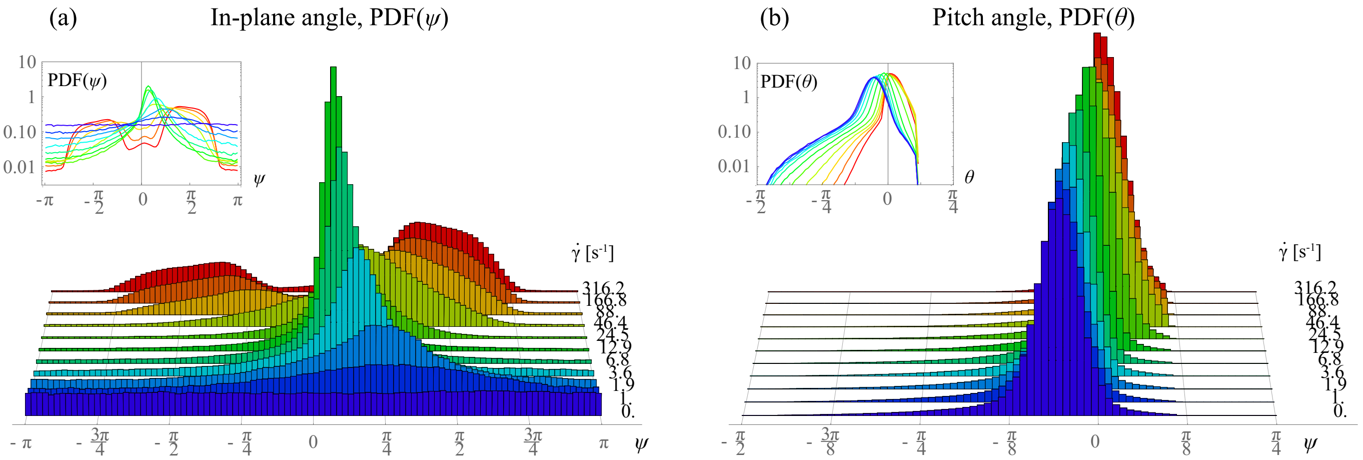

Supplementary Figure 2. Effect of tumbling. Shown is the distribution of the in-plane angle ψ for tumbling bacteria (see Methods §7) for different shear rates $\dot{\gamma}$. For comparison, the same distributions without tumbling are shown in Fig. 5(a,b).

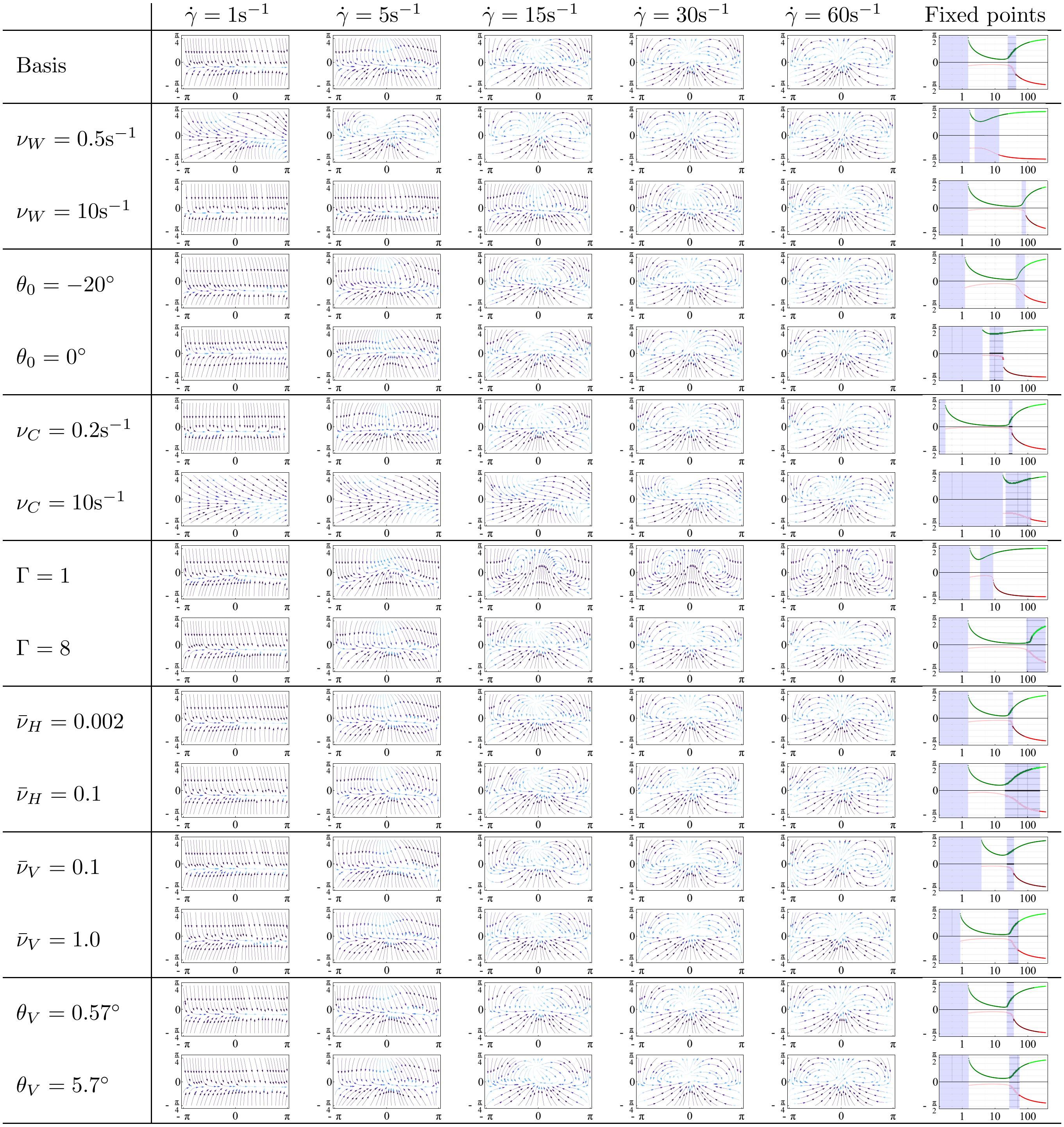

Supplementary Figure 3. Robustness of the model with respect to parameter variations. The first row repeats our results for the basis parameter set, and subsequent rows show how these change when parameters are varied one by one, keeping the other parameters from the basis set. In the first five columns we show the bacterial dynamics in $\phi - \theta$ phase space at the shear rates $\dot{\gamma} = 1, 5, 15, 30, 60\text{s}^{-1}$, respectively. The last column also gives the rheotaxis diagram of the equilibrium in-plane angle, ψ^* as a function of $\dot{\gamma}$ [s^{-1}], obtained numerically from the deterministic model, as in Fig. 5(c). Here light red/green points mark an unstable fixed point, dark colours indicate a stable fixed point, and ordinary red/green points mark a stable limit cycle. The four rheotaxis regimes (*I*-*IV*) are marked with blue and white areas.
